# Examining the pathway to specialist care for children and young people with late presentation of chronic kidney disease in the UK: a qualitative study

**DOI:** 10.1136/bmjopen-2024-096266

**Published:** 2025-11-09

**Authors:** Lucy Plumb, Manish Sinha, Matthew J Ridd, Fergus Caskey, Yoav Ben-Shlomo, Amanda Owen-Smith

**Affiliations:** 1 Population Health Sciences, University of Bristol Faculty of Health and Life Sciences, Bristol, UK; 2 UK Renal Registry, Bristol, UK; 3 Department of Paediatric Nephrology, Evelina London Children’s Hospital, London, UK; 4 King’s College London, London, UK

**Keywords:** Paediatric nephrology, PAEDIATRICS, QUALITATIVE RESEARCH, Chronic renal failure

## Abstract

**Abstract:**

**Objective:**

Detecting chronic kidney disease (CKD) early can provide opportunities to optimise native kidney function, prevent further decline and plan for timely kidney transplantation if required. Understanding how children are found to have kidney disease and present to specialist kidney care may help tailor interventions to support a timelier diagnosis. The aim of this study was to examine the pathway to specialist care for UK children who present late to nephrology with advanced CKD (requiring kidney replacement therapy within 90 days of first nephrology review) to determine whether there are modifiable aspects to presentation and diagnosis.

**Design:**

Semi-structured, in-depth qualitative study. A topic guide based on the theoretical framework of health behaviour by Scott *et al*, *The Model of Pathways to Treatment,* was developed to capture differences in symptom appraisal and help-seeking before reaching nephrology care.

**Setting:**

UK paediatric nephrology units (n*=*4) between December 2017 and December 2020.

**Participants:**

Children and young people who experienced a late presentation of CKD and their parents/carers.

**Results:**

Twenty-two participants participated across 19 interviews: seven children (two male, median age 16, IQR 13–17.5 years) and 15 parents. A typology of presentation to healthcare was identified: commonly, families reported repeated cycles of primary care help-seeking before onward referral to specialist care, although long appraisal intervals were also noted. In all cases, secondary care referral led to onward nephrology care involvement. Narratives highlighted that not all cases of late presentation could be avoided.

**Conclusions:**

A typology of symptom appraisal and help-seeking can inform interventions to improve CKD detection. Interventions that support symptom appraisal and consideration of targeted CKD testing in children may help reduce appraisal and help-seeking intervals, respectively.

**STRENGTHS AND LIMITATIONS OF THIS STUDY**

- In-depth qualitative interviewing is a useful and appropriate methodological approach to understand individuals’ experiences and the complex social processes underlying the late presentation of chronic kidney disease.
- This was a multicentre study with participant representation from different ethnicities, ages and socioeconomic backgrounds, therefore, findings are likely to be generalisable to the broader UK population.
- Interviews were conducted with five children independently of their parents; this enabled triangulation of accounts and greater insight into parent-child interactions relating to symptom appraisal and help-seeking.
- Narratives of the pathway to diagnosis and specialist kidney care were obtained retrospectively and therefore may be influenced by subsequent events and reflections.
- Study findings lack comparison with accounts from timely presenting families, to understand where similarities and differences in experiences exist.

## Introduction

 Chronic kidney disease (CKD) in childhood is associated with significant health implications including impaired growth,^1^ bone disease^2^ and anaemia, while progression to kidney failure correlates with accelerated cardiovascular morbidity,^3^,^5^ shortened life expectancy^6^ and worse psychosocial outcomes compared with peers.^7 8^ Additionally, CKD treatment is costly given its associations with higher all-cause mortality, excess cardiovascular morbidity and healthcare requirements for the treatment of kidney failure.^9^

In recent years, public health campaigns have emphasised the importance of early detection in preventing excess morbidity and mortality and mitigating healthcare costs associated with disease progression. For children, detection of those at risk, or in the early stages of CKD, allows them to benefit from interventions to slow disease progression and optimise growth, as well as plan for future treatments such as kidney transplantation, the preferred and most cost-effective kidney replacement therapy (KRT).^10 11^ However, with an estimated incidence of up to 18 per million of the age-related population,^12^ a timely diagnosis for a rare disease is challenging. Globally, high proportions of children with CKD first present to specialist nephrology care with advanced disease, most often in kidney failure.^13^ In the UK, a quarter of children requiring long-term KRT present late to specialist care, within 90 days of starting KRT.^14^ It is not fully understood how late presentation in childhood CKD arises or to what extent it may be preventable. Studying the lived pathways to diagnosis for children with chronic conditions such as CKD may help identify modifiable targets for intervention to improve timely diagnosis and treatment.^15^ The aim of this study was to obtain an in-depth understanding of the pathway to specialist kidney care for children and young people who first present to nephrology services with advanced CKD.

## Methods

### Study design

In-depth, semi-structured interviews were conducted with children and young people aged 6–18 years or parents/carers of children aged 1–18 years who had experienced a late presentation of their kidney disease within the last 5 years. Late presentation was defined as needing to commence long-term KRT within 90 days of the first paediatric nephrology review.^16^ Identification and recruitment of eligible participants occurred at paediatric nephrology units across England between December 2017 and December 2020. Individuals lacking the capacity to consent or recall events prior to diagnosis were excluded.

### Sampling strategy

A purposive, theoretically informed approach to sampling was taken. Clinical and research leads at each recruiting site were asked to identify eligible participants across the paediatric age spectrum, to examine the impact of age on symptom recognition, parental interactions and help-seeking behaviour. For diversity of sampling, identification of patients from different ethnic backgrounds, as well as parents of both sexes, was also encouraged.

### Recruitment and consent

After expressing an interest in participation, eligible participants were contacted by the study lead by email or telephone. Participants were provided with information about the study prior to the interview. Informed consent was taken from participants aged 16 years or over; informed assent was obtained for children under 16 years, along with written consent from a parent or guardian. All interviews were conducted in person except one, which took place via encrypted video call. Interviews took place at the participant’s home or in a private clinical room at their local nephrology unit, depending on participant preference. All interviews were conducted by LP (qualifications BMedSci BMBS MRCPCH), a female doctoral researcher at the time of the study. As a clinical doctor in the NHS before starting her doctoral fellowship, LP had trained in two of the nephrology units that recruited participants to the study, but had not undertaken regular clinical duties for almost 2 years prior to the study start. For this reason, LP introduced herself as a ‘researcher’ from the University of Bristol Medical School, although she offered additional information about her clinical background as requested.

### Topic guide

A topic guide (online supplemental file 1) informed by a theoretical framework proposed by Scott *et al,* The Model of Pathways to Treatment, was used to capture participants’ lived experiences of late presentation: from first identifying a problem to seeking help and reaching specialist nephrology care.^15^ In this model, the pathway to treatment is considered as two intervals before presentation to healthcare: the appraisal interval (from detection of a bodily change to deciding to seek medical help) and the help-seeking interval (from deciding to seek medical help to first consultation). There is a further diagnostic interval from the first medical consultation to formal diagnosis. The topic guide was developed and pilot tested with patient representatives and applied flexibly in interviews.

### Data analysis

Interviews were recorded and professionally transcribed. A thematic analysis approach was used to analyse the data.^17^ QSR NVivo V.12 was used to aid coding and organisation of the data. All transcripts were coded by LP, with a sample double-coded by an experienced qualitative researcher (AO-S) to enhance reliability. Data analysis was inductive and iterative, using the constant comparative method to review and compare codes, develop themes, and refine and integrate themes where possible, thus facilitating theoretical development.^18^ Data saturation was determined based on discussions between LP and AO-S. Participants were not involved in the analysis or interpretation of interview data. Findings are reported in accordance with the Consolidated criteria for reporting qualitative studies.^19^

###  Ethical approval

This study received Health Research Authority and West Midlands- Coventry and Warwickshire Research Ethics Committee approval: reference 17/WM/0310.

### Patient and public involvement

Patients and their families were involved in identifying the research question, study design, and creating and piloting the topic guide. Once the findings are published, participants will be informed of the results through a summary of the findings.

## Results

In total, 27 eligible participants were identified across five nephrology units in England. Four participants were not contactable following initial expression of interest; one declined to participate. The remaining 22 participants (seven children and 15 parents from 13 families, table 1) participated across 19 interviews. Interviews with child participants lasted 36–71 min; parent interviews lasted 43–120 min. Seven children (28.6% male) were interviewed independently; two children participated with a parent present. The median age of children participating was 16 (13–17.5) years. Demographic and clinical characteristics of participants are presented in table 2. Of the participating children and young people, one (family #2) had a historical diagnosis of acute kidney injury (AKI) at birth and had been under paediatric follow-up in early childhood. Three children and young people had co-existing medical conditions and were under paediatric care: two participants had neurodevelopmental diagnoses (families #6 and #8), while a third child had an underlying syndromal diagnosis and was under the care of several paediatric specialists at the time of her kidney diagnosis (family #10). She had been screened for kidney disease in early childhood. One child had been born prematurely (<30 weeks gestation, family #6).

**Table 1:** Coding key for participants, grouped by family (n*=*13)

Family number	Parent number	CYP number
1*	Parent #1a, female Parent #1b, male	CYP #1, female
2	Parent #2, female	CYP #2, male
3	Parent #3, female	CYP #3, female
4	Parent #4, female	CYP #4, female
5	Parent #5, female	CYP #5, female
6*	Parent #6a, female Parent #6b, male	CYP #6, female
7		CYP #7, male
8	Parent #8, female	
9	Parent #9, female	
10	Parent #10, female	
11	Parent #11, female	
12*†	Parent #12a, male Parent #12b, female	
13	Parent #13, female	

* Parents interviewed as a dyad.

† Parents interviewed via interpreter.

CYP, children or young person.

**Table 2:** Demographics of participants, by family (n*=*13)

Variable	n (%)
**Ethnic group** Asian Black White Other	2 (15.4) 0 10 (76.9) 1 (7.7)
**IMD quintile (based on residential postcode)** 1—most deprived 2 3 4 5—least deprived	2 (15.4) 3 (23.1) 1 (7.7) 5 (38.5) 2 (15.4)
Primary kidney disease of child* Unknown Glomerular disease Systemic diseases affecting kidney Miscellaneous Familial/hereditary Tubulointerstitial disease†	4 (30.8) 3 (23.1) 3 (23.1) 1 (7.7) 1 (7.7) 1 (7.7)
**Median duration since first nephrology review (IQR)**	22 (15–42) months
Occupation of interviewed parent/carer‡ Professional Technicians and associate professionals Clerical support workers Service and sales workers Unemployed Not known	3 1 2 1 7 1

* Categorised using European Renal Association classification.^11^

† Disorders comprising congenital anomalies of the kidneys and urinary tract.

‡ Occupations classified using the International Standard Classification of Occupations (https://ilostat.ilo.org/methods/concepts-and-definitions/classification-occupation/); two participants currently unemployed reported previous professional occupations before their child’s diagnosis.

IMD, Index of Multiple Deprivation.

Each participant was asked to describe approximate timings from first noticing symptoms until reaching specialist kidney care. Where participants came from the same family, parents tended to report shorter durations of symptoms than children.

### Symptom appraisal

All participants described identifying symptoms prior to presentation to health services (table 3). In almost all cases, symptoms were initially appraised as no cause for concern. Participants evaluated symptoms in light of their (or their child’s) age, development and routine, leading to normalisation of symptoms, or attributed symptoms to benign conditions, which was influenced by a perceived lack of supporting evidence for serious pathology (box 1). Symptom appraisal and the decision to seek medical review were influenced by external sources for both parents and children, including friends, family and feedback (or lack thereof) from school (box 1). While most children reported identifying symptoms themselves, the extent to which they communicated these symptoms with parents varied. In four cases, symptom appraisal was hindered by disrupted communication between child and parent (box 1). Symptoms that were not normalised by participants were associated with short appraisal intervals and immediate help-seeking.

**Table 3:** Symptoms described by participants and their reported frequency (by family, n*=*13)

Symptom	Number citing symptom	Illustrative quotes
Poor appetite +/− weight loss Poor growth	12	*‘Then I’d noticed she started to lose weight, and I thought, ‘That’s the weight that sometimes children gain in primary school, and then when they get older, they’re more active, they’re just losing weight. Like that ‘puppy fat’ sort of thing. She was getting too thin, I thought’ (Parent #6a, female*) *‘I was losing weight as well and my trousers that I normally wore were falling as I was walking back from the doctors’ (CYP #7, male*)
Fatigue	10	*‘I used to go there (parent’s house) fortnightly, and every time I went there, I’d go on the sofa and be fast asleep and he’d be like, ‘What’s going on? ‘Why are you always tired?’ I was just always tired’ (CYP #2, male*)
Lethargy	8	*‘She just seemed very, sort of, lethargic, didn’t really have a great deal of ‘oomph’.’ (Parent #5, female*) *‘*(*He coughs) most of the night, he doesn’t have energy like his peers, fatigue and the nausea, stuff like that’ (Parent #12a, male*) *‘I remember I was in the same spot on the sofa all day just… yeah, basically not being able to move that much and I didn’t really know what was going on’ (CYP #7, male*)
Nausea +/− vomiting	7	*‘I started to feel really, really unwell and then during my second lesson, I just chucked up and I felt really embarrassed and it kept happening’ (CYP #6, female*) *‘In December, I remember him vomiting, losing weight. I took him to the general practitioner and it was just seen as something that would clear up by the summer once he’d got his exams out of the way’ (Parent #9, female*) *‘(Child) would vomit; I mean, projectile vomit. As soon as it was in, she would vomit, and it was constantly changing, crying baby’ (Parent #13, female*)
Cough	4	*‘Things like she’d wake up in the morning and she’d spend 1 hour in the toilet coughing and vomiting, coughing and vomiting’ (Parent #3, female*) *‘She had this cough, and she really was tired. She wasn’t sleeping at night with it. It kept waking her up, she said, coughing. But she didn’t say there was anything else. She just said it was a cough, but I didn’t like the way that she looked’ (Parent #5, female*)
Swelling	4	*‘Her feet were like elephant’s feet, literally’ (Parent #10, female*)
Itching	3	*‘She would scratch herself so badly that she would literally look like a cat’s gone at her’ (Parent #13, female*) *‘And then she started getting some rashes on her legs. Again. I just thought maybe it’s, you know, from clothing irritating her legs, heat rash, scratching a bit too much’ (Parent #4, female*)
Pallor	3	*‘I was pale. Even my teachers were saying, ‘Do you feel all right?’ (CYP #2, male*) *‘My teacher told me what my headmistress was saying, that apparently I was very shaky and very pale’ (CYP #7, male*)
Bad breath or odour	3	*‘She used to have this smell coming from her mouth, and I took her to the dentist just in case there was something wrong with her gums or her teeth, and he said no fine, she’s got nothing wrong with her. And it was a specific smell, and her (siblings) used to be really mean and say, oh you know, she’s got bad breath and this sort of thing, and it made her a bit paranoid’ (Parent #4, female*) *‘And I’d noticed that she looked quite swollen in her face, and she’d got quite a smell from her breath. It was a really strange smell. I’d never smelt it before. It was almost like a stale urine type of smell’ (Parent #8, female*)
Visual problems Back pain Dry skin/rash/hair Generally unwell Leg pain/muscle cramps Shaky/felt cold	Reported by two families each	*‘He was just saying that he couldn’t see very well. He spends an awful lot of time on the computer, so I put it down to that really, and I didn’t think there was a problem with his eyesight’ (Parent #9, female*)

CYP, children or young person.

Box 3 **Factors contributing to late presentation, from parents’ perspectives**

**Child and family factors**

- Increasing child independence with age
  - *‘She would tell me about the rashes on her legs because most of… at her age obviously she baths herself and she was covered up quite a lot’ (Parent #4, female*)
- Age/behaviour influenced interpretation of symptoms
  - *‘If, say, (child) was six or something, that may have been different, or an adult? Because an adult, it’s not normal behaviour for an adult just to want to lie down… You know something is wrong. But for a teenager, you think, ‘They want to stay in their room’ (Parent #6a, female*)
  - *‘If (child’s sibling) suddenly started to say to me, ‘I’m tired and I don’t want to go to school, my back’s hurting.’ I would be in the doctor’s saying, ‘This isn’t the nature of this child, that’s not right, there’s something wrong.’ (Child*)*’s a child with behavioural problems, so for 5 years she’s hated school and I’ve heard every excuse’ (Parent #10, female*)

**Disease factors—‘Hidden in plain sight’**

- Insidious symptoms
  - *‘What probably doesn’t help with the diagnosis of the children is that they live with it, they grow with these conditions and they don’t know any different. (Child) had no idea she was ill and she never presented as ill’ (Parent #1b, male*)
- Symptoms considered in isolation/not recognised given rarity
  - *‘And then we found out that the smell that was coming from her mouth was a distinctive smell but a lot of general practitioners (GPs) obviously don’t come across people like (child) very often, so they wouldn’t know that smell. But had they known that smell then perhaps things would’ve got picked up a bit sooner, I don’t know’ (Parent #4, female*)
- Symptoms not conforming to what is ‘expected’
  - *‘It was really shocking to hear that both kidneys are not working and the doctor was also amazed to say that ‘Why she’s not having swelling?’ (Parent #3, female*)

**Healthcare factors**

- Failure to communicate between professionals
  - *‘She just was under so many different people, that maybe that was the problem. They were all looking at their own speciality and maybe not looking outside the box’ (Parent #8, female*)
- Perceived reluctance to test children
  - *‘Even when I requested one from the GP and said ‘Why can’t you do a blood test?’ He said they normally don’t do for the young children until there is a big concern’ (Parent #3, female*)

**Lack of social (background) knowledge**

- Lack of knowledge about kidney function
  - *‘When you realise, when you obviously learn what the kidneys do, you wish you’d known it beforehand because you would know the signs. ‘If you knew what the signs were and what they do, you would know.’ ‘Now, to me, it was all there. Now I know more about kidney disease and how it can affect growth, it was just staring us in the face, really’ (Parent #8, female*)
- Disease misconceptions
  - *‘And especially in our case because there’s no history of it, so things only get checked normally if you’ve got a history of something. I don’t know’ (Parent #4, female*)
  - *‘You know, this dialysis term I only heard for old people who had diabetes. ‘I’d never ever come across any children with dialysis, you know, kidney failure, until I came here’ (Parent #3, female*)

Box 1 **Participant quotations by theme**

**Symptom appraisal**

- Normalisation of symptoms
  - *‘I just thought, you know, she’s growing up, she’s doing a lot of exercise at school, just general aches and pains, didn’t think anything of it’ (Parent #4, female*)
  - *‘He was sleeping in the evening… That was worrying me. I thought, ‘Stop being on your phone and your computer and sleep’ (Parent #2, female)*
  - *‘Because I used to do PE a lot and running and stuff, me and my Mum just thought they were growing pains and just leg pains after I had been running or doing a lot of exercise’ (CYP #4, female*)
  - *‘I talked to you about it, didn’t I (mum*)*? And you’d tell Dad. All I just said was that I felt really unwell and you said, ‘Quit being lazy, go to school’. Because I don’t think you expected it, did you?’ (CYP #6, female*)
- Lack of supporting evidence to suggest serious pathology
  - *‘We don’t have any family history of kidney issues so we never knew all these things …we thought maybe you’re not eating quite well and that makes you sick in the morning, that’s all we thought…’ (Parent #3, female*)
  - *‘The school had never said, ‘*(*child) is falling asleep in class’, or…‘we’ve noticed something unusual with (child*)*’…and she didn’t look sick, which is why when she was just tired without the other symptoms, I just thought, ‘She just wants to not do anything’ (Parent #6, female*)
- Influence from external sources
  - *‘I told my friends and they’re like, yes I’m being sick in a morning as well. It’s normal, so I thought it was normal because my friends were being like, yeah I’m always sick in the morning, um yes I’m always tired, so I thought it was something normal that everyone does, so I didn’t think much of it’ (CYP #3, female)*
  - *‘So (friend) came round and he’d worked in Emergency Departments for 20 odd years and she was asleep by this time and he looked at her and he was like ‘she’s definitely not right’.’ (Parent #1a, female*)
  - *‘My mum took me to the general practitioner (GP) to get my bloods tested because my teacher said for me to go to the doctor’ (CYP #7, male*)
  - *‘(Wife) has a friend…who is a pharmacist, she showed her (blood test request from GP) and she… said, ‘right away you have to do that’ (Parent #12, male*)
- Disrupted communication between child and parent
  - *‘She kept asking if I was getting sleep and stuff. I kept saying I was fine. I’ve never really liked hospitals, so I’ve never said that I felt ill. I’ve always said I felt fine’ (CYP #2, male*)
  - *‘When he started sleeping, I noticed that straightaway…When I asked him, ‘Do you feel all right, (child*)*?’ ‘Yeah, I’m all right.’ ‘Why are you going upstairs?’ ‘I’m going to watch telly.’ He had an answer for everything’ (Parent #2, female*)
  - *‘I thought it was something normal that everyone does so I didn’t think much of it. I was like maybe everyone’s doing this but they’re not showing it, so I was like I’ll just hide it or something like that’ (CYP #3, female*)
  - *‘No, I don’t think so. I think at the time, we didn’t really talk that much. I normally did my homework in my room or I was out with friends, and she (Mum) just got home from work and she did her own thing. So, we didn’t really talk that much’ (CYP #5, female*)
  - *‘She never said that she couldn’t breathe, that she had trouble breathing. She never mentioned that at all and I think that’s the bit that I really struggled with…I’ll always wonder should I have questioned her’ (Parent #5, female*)

**Tipping point for seeking medical review**

- Physical change causing disruption to normal activity
  - *‘She couldn’t stand very well and she was 45 kg and all of a sudden she’d gone down to 36–37 kg in a month period’ (Parent #3, female*)
  - *‘She was always wanting to play with her friends and being on her bike, and she didn’t want to do that, so when she stopped wanting to go out, I thought this is not her’ (Parent #4, female*)
  - *‘By that point, she looked dreadful. She was so swollen. There was no getting away from, ‘Oh I think it’s just a virus.’ By that point, you could just see how unwell she was’ (Parent #8, female*)
- Change in symptom pattern making previous assertions unlikely
  - *‘I thought she’d just gone too slim, and again, but didn’t link that until the headaches and the vomiting. Then I thought, ‘Maybe it’s not a virus’ (Parent #6, female*)
  - *‘I took him to the GP a couple of times but because of what was happening in his life, that is, GCSEs, it was just put down to that… When it came to the summer of that year, he wasn’t getting any better; he was getting worse, actually. He was still vomiting’ (Parent #9, female*)
- Lack of resolution
  - *‘I think it was the fact that she just didn’t seem to improve. You know that viruses can knock you for six for a few days, but after a couple of weeks, she just wasn’t picking up’ (Parent #8, female*)

CYP, children or young person; PE, physical education.

Box 2 **Typology of diagnostic pathway**

**Acute presentation: Immediate appraisal and help-seeking intervals**

- *‘When she woke up in the night, screaming in pain and she said, ‘Mummy, I’m not feeling well, my tummy’s hurting, I need the toilet’. And then when we saw what she passed through, it was like breathtaking really. You were like, oh my god, oh my god’ (Parent #11, female*)

**Short appraisal, help-seeking and diagnostic intervals**

- *Female parent: ‘So (friend) came round and he’d worked in Emergency Departments for 20 odd years and she was asleep by this time and he looked at her and he was like ‘she’s definitely not right’*
*Male parent: He said she’s oedemic straight away, she’s oedemic. Look at her legs. She’s oedemic*
  *Female parent: She’s clearly reacting to something, but I don’t know what it is. ‘Take her back to the doctors’ (Parents #1a and #1b*)
- *‘And then, they (GP surgery) rang me and asked me whether she’d improved over the last days since they’d seen her and I said, ‘No, she still seems quite unwell.’ And I’d noticed that she looked quite swollen in her face and she’d gotten a smell from her breath. It was a really strange smell. I’d never smelt it before. It was almost like a stale urine type of smell. When I took her back to the GP, I said these have sort of just happened over the last couple of days… Then the GP said we think you need to go to the Children’s Assessment Unit at (local hospital) for further tests’ (Parent #8, female*)

**Long appraisal interval, short help-seeking and diagnostic intervals**

- *‘Well, to be honest, sort of at the start, I thought it was normal to feel like that? …To be not very hungry at all. I mean, I thought it was just a stage that kind of everyone went through, to be honest’ (CYP #7, male*)
- *‘But then he started coming home from school and he’d come in and go upstairs and go to sleep. He’d get up in the morning and be so pale, and I was watching and then (my partner*) *noticed on his legs he had all bruises, and he asked him and he said, ‘Oh, me and my mates were just playing.’ We left it at that’ (Parent #2, female*)
- *‘So, it was only when the tiredness, the rash on her legs, the bags under her eyes that I thought well let’s… it could all be linked or we could just try and get it all dealt with at one appointment. Because I’m not one of these parents who is constantly taking my child to the doctors because of a cold or just general aches and pains. I work in a doctor’s surgery so I know how many wasted appointments there are, so I try not to waste the doctor’s time if I can help it’ (Parent #4, female*)

**Repeated appraisal and help-seeking intervals**

- *‘I took him to the GP a couple of times but because of what was happening in his life, that is, GCSEs, it was just put down to that’ (Parent #9, female*)
- *‘The GPs were very blasé; they would just look at me and they would just be like, ‘Oh, you’re back again. She’s fine. She’s fine’ (Parent #13, female*)
- *‘So, I was seeing (different GPs) and they were saying … they were asking me ‘Do you have any concerns?’ And I said, ‘I have the concerns that she’s vomiting, she’s coughing’ and ‘Do you have a temperature?’ and I said ‘No’. It’s the same time… same questions’ (Parent #3, female*)
- *‘(GP) was like maybe she’s just depressed because (of family incident) …but I was like it’s nothing to do with (incident*)*. It’s just that I’m not feeling well’ (CYP #3, female*)
- *‘I was like, ‘My baby is clearly really ill. I’m here all the time and I’m literally begging you lot. ‘Something’s not right.’ Then I said to her—I remember it so well—I said to her, ‘I was just wondering if there’s something wrong inside. Maybe it’s her organs. Can you do a scan or something?’’ (Parent #13, female*)
- *‘I’m blaming myself and my wife because we should have been more firm taking him (for a blood test) and maybe that would have been a totally different story’ (Parent #12, male*)

CYP, children or young person; GP, general practitioner.

### Tipping point for seeking healthcare review

Participants sought medical help when symptoms no longer conformed to prior judgements, could no longer be normalised, or attributed to benign conditions (box 1).^5^,^7^ While children reported seeking help from friends, parents were the primary drivers for seeking formal healthcare review. Tipping points for seeking help included when a physical change was noticed, particularly one that disrupted normal activity; a change in symptom pattern which made previous assertions less plausible; or a lack of resolution.

### Typology of the diagnostic pathway

From participant accounts, four patterns of symptom appraisal and help-seeking were identified. These did not appear to be related to either age or sex. Except for one acute presentation associated with haemolytic uraemic syndrome, presentation did not appear to be related to subsequent kidney diagnosis (table 4, box 2).

**Table 4:** Presentation pattern by family and underlying kidney diagnosis

Family number	Pattern of presentation	Child’s kidney disease diagnosis
1	Short appraisal, help-seeking and diagnostic intervals	Postinfectious glomerulonephritis on background of small kidneys
2	Long appraisal interval, short help-seeking and diagnostic intervals	Kidney impairment (reported to be after perinatal insult)
3	Repeated appraisal and help-seeking intervals	ANCA vasculitis
4	Long appraisal interval, short help-seeking and diagnostic intervals	Unknown
5	Short appraisal, help-seeking and diagnostic intervals	Goodpasture’s syndrome
6	Repeated appraisal and help-seeking intervals	Unknown
7	Long appraisal interval, short help-seeking and diagnostic intervals	Nephronophthisis
8	Short appraisal, help-seeking and diagnostic intervals	Kidney dysplasia
9	Repeated appraisal and help-seeking intervals	Unknown
10	Short appraisal, help-seeking and diagnostic intervals	Unknown
11	Acute presentation	E. coli associated haemolytic uraemic syndrome
12	Repeated appraisal and help-seeking intervals	Focal segmental glomerulosclerosis
13	Repeated appraisal and help-seeking intervals	Congenital nephrotic syndrome

ANCA, anti-neutrophil cytoplasmic antibody.

1. *Acute presentation: immediate appraisal and help-seeking intervals*

This pattern of presentation, identified in one case, was characterised by the rapid onset of symptoms with urgent presentation to health services. One parent described a sudden onset of acute symptoms (pain and bloody diarrhoea) in her child, which were sufficiently alarming as to prompt urgent medical review via the emergency department, leading to admission and nephrology referral. Symptoms also occurred in the middle of the night, which may have played a role in the source of help-seeking.

1. *Short appraisal, help-seeking and diagnostic intervals*

Four families described a relatively short symptom appraisal interval (days to weeks) before seeking medical help. In each case, families described symptoms that were initially attributed to an alternative diagnosis. In three cases, parents were prompted to return for further medical review within days following deterioration in their child’s condition; one family sought confirmation of concerns from a family friend before re-seeking medical help. For the fourth family, urine testing at the initial medical review identified significant proteinuria, prompting onward referral to the hospital.

1. *Long appraisal interval, short help-seeking and diagnostic intervals*

Participants from three families described symptoms noticed over periods of months to years. As in other cases, symptoms were initially normalised. Symptoms were seemingly indolent and therefore accommodated into daily life without raising concern until their progression prompted re-appraisal; children were also peri-pubertal, therefore participants frequently ascribed symptoms to puberty or ‘normal’ teenage behaviour. Two of the young people who reported evading parental enquiries about symptoms described long appraisal intervals, which triangulated with parental accounts. For all families describing this presentation, the initial healthcare review prompted investigation (blood tests) and referral to onward secondary care.

1. *Repeated appraisal and help-seeking cycles*

This presentation, identified in the accounts from five families, was characterised by an iterative pattern of appraising symptoms and seeking healthcare review for symptoms later attributed to CKD. Many participants were initially reassured, treated for alternative diagnoses or advised to observe symptoms. In three cases, medical review failed to fully allay concerns regarding their child’s symptoms, with increasing frustration noted from parental accounts when concerns were dismissed or failed to elicit action. In these narratives, a sense of not being listened to led several parents to seek different opinions, often from different general practitioners (GPs). Concerns were exacerbated by the same line of questioning at each consultation, which was perceived by participants as a failure to consider persistent symptoms in a different light. For three families with older children, GPs were perceived as not having listened to or verified their assessments with the child directly. Three parents who attended the GP more than once before referral described being increasingly proactive at later consultations by suggesting investigations or treatment during later GP encounters.

Two family accounts experiencing this presentation type did not describe frustration or dissatisfaction with the GP; in one case, a father blamed himself for not agreeing to a blood test, while in the second narrative, the family considered the evolution of symptoms and resultant management to be appropriate prior to referral. Referral to secondary and subsequent nephrology care was subsequently initiated in four cases by a blood test and following high blood pressure readings in another.

### Factors implicated in late presentation

Box 3 presents factors described by parent participants that they perceived hindered the timely presentation of their child’s kidney condition. These included social knowledge, disease, healthcare and child and family factors.

## Discussion

This study investigated the sequence of events leading to diagnosis and specialist nephrology care for children with advanced CKD and offers novel insights into experiences of late presentation. From participant accounts, we identified a typology of appraisal and help-seeking, which may help understand how interventions to improve the timeliness of help-seeking and referral can be developed.^15^ For many families interviewed, there appeared to be possible opportunities for earlier detection.

While a number of qualitative studies have explored the experiences and perceptions of children and young people with CKD^20^,^24^ and their families,^25^ little focus is placed on the pathway to diagnosis and specialist care. We identified that in all families sampled, symptoms attributed to CKD were present before diagnosis and recognised by both children and parents, which aligns with our previous quantitative work.^26^ For several families, appraisal of symptoms was influenced by disease (quality, nature, pattern) and child (age, developmental stage, daily routine) factors. Many of the factors identified as the ‘tipping point’ for seeking healthcare review in this study correlate with those cited within the broader help-seeking literature.^27^ In addition, we identified that a change in symptoms, which rendered previous judgements implausible, prompted participants to seek help, thus highlighting the dynamic and iterative process of symptom appraisal. Interestingly, many families gave weight to a lack of family history when appraising symptoms, believing kidney disease to be a hereditary condition. Similar beliefs have been demonstrated among cancer and CKD patients,^28 29^ highlighting how understanding of the genetic basis of disease has shaped lay assessments of illness risk. Additionally, concerns (or lack thereof) expressed by the school influenced several families’ actions, highlighting the importance of communication and sharing concerns to inform symptom appraisal. Triangulation of accounts between parents and their children also demonstrated a mutual dependence on sharing concerns to adequately evaluate symptoms. As many parents cited child feedback as a component of symptom appraisal, a lack of information or concern from the young person may have distorted parental assessments, leading to longer appraisal intervals. Child reporting of symptoms to caregivers has been postulated as a key factor influencing the pathway to treatment for other chronic conditions and, as such, may help accelerate progression through the diagnostic pathway.^30^

We identified four patterns of presentation which may have implications for future interventions aimed at reducing time to diagnosis for children with advanced CKD. In the sample of families interviewed, acute presentation represented AKI which failed to recover, leading to dialysis dependence. Most children experiencing a semi-acute presentation had underlying glomerular or unknown kidney disorders, which may represent rapidly progressive disease that was not amenable to intervention. However, we also noted that children with these conditions also experienced other patterns of presentation, emphasising the importance of symptom recognition and targeted kidney testing when these are present.

The most common presentation was one of repeated appraisal and help-seeking, suggesting delays to diagnosis may occur due to healthcare factors and are thus potentially modifiable. Key in this pattern of presentation was the relationship between the parent and primary care clinician in shared decision making. Many parents described their frustration at a perceived failure to acknowledge concerns and consequently took an increasingly proactive stance to influence the consultation outcome at subsequent attendances. These findings emphasise the responsibility felt by parents to advocate for their child and correlate with findings from cancer studies, where persistence and ‘taking the initiative’ are employed when concerns persist.^31 32^ Furthermore, acknowledging parental concern has also been shown to play a role in identifying serious illness.^33^ Our findings highlight the importance of actively involving families in primary care consultations to ensure concerns are adequately addressed, thus avoiding threats to parental credibility. Recognising also that a CKD diagnosis is a ‘once-in-a-lifetime’ diagnosis, interventions such as a clinical prediction model embedded in clinical systems could help identify those at highest risk of disease and support use of non-invasive or point-of-care testing, thus avoiding the need for specific knowledge or training in a rare condition.

For patients who experienced long appraisal periods, interventions which improve lay knowledge of kidney disease and associated symptomatology may help reduce this interval. Awareness of kidney disease and function among the general population is recognised to be low^34^ and was cited as a barrier to diagnosis by participants in this study. Raising awareness of symptoms associated with kidney function decline could arm families with the requisite knowledge to consider timelier medical review, particularly in light of a child’s age and other risk factors. Given the rarity of childhood CKD, it is unclear whether such campaigns would prove successful in improving timely detection; however, a similar campaign for childhood brain tumours was successful in reducing the total diagnostic interval,^35^ suggesting this may be feasible. Such campaigns may also help care professionals by increasing exposure to conditions they are unlikely to encounter regularly.

The study identified that for most families, there was no known underlying kidney disease and that late *referral* to nephrology services was not implicated in late presentation. However, three children had potential risk factors for the development of CKD in the form of an underlying syndromal disorder, AKI and prematurity, respectively, raising the question of whether targeted monitoring of those in known at-risk categories may have led to earlier diagnosis and intervention. Similarly, as the incidence of kidney failure (and thus late presentation) is highest in adolescence,^14 16^ consideration of targeted kidney testing in the context of a child’s age when key symptoms are present may support earlier detection. With increasing epidemiological evidence of childhood risk factors implicated in long-term kidney impairment,^35^,^37^ establishing interventions to identify CKD and prevent progression in those at risk may be of long-term public health and cost-benefit. ^38^ This study benefits from its multicentre design, inclusion of children and parents, which allowed triangulation of key themes, and its in-depth, semi-structured approach, which resulted in rich narratives while allowing participants to flexibly share their experiences. It must be acknowledged, however, that participant accounts were obtained retrospectively and therefore may have been influenced by subsequent events and reflections. Memory recall may have also been affected, although the 2-year timeframe was chosen following discussion with patient representatives, given the significance of the event. It is also important to acknowledge that these findings represent the accounts of late-presenting families and lack comparison with timely-presenting families. This study did not include children under 1 year at diagnosis, and therefore, it is unclear whether findings are generalisable to this cohort, who may have greater exposure to health professionals. Although this study was conducted in England only, given similarities in healthcare provision, we expect these findings to be applicable to devolved UK nations and potentially other countries with publicly funded healthcare systems.

In conclusion, this is the first study to examine, in detail, the experiences of children, young people and families of the pathway to diagnosis for those with late-presenting CKD. Strategies supporting symptom appraisal and consideration of targeted testing in CKD, particularly following repeated attendance, may help reduce time to diagnosis. Whether such strategies are cost-effective in managing this rare but lifelong, expensive condition, however, requires further investigation.

## Supplementary material

10.1136/bmjopen-2024-096266 online supplemental file 1

## Data Availability

Data are available upon reasonable request.

## References

[1] Seikaly MG, Salhab N, Gipson D (2006). Stature in children with chronic kidney disease: analysis of NAPRTCS database. *Pediatr Nephrol*.

[2] Bacchetta J, Harambat J, Cochat P (2012). The consequences of chronic kidney disease on bone metabolism and growth in children. Nephrol Dial Transplant.

[3] Litwin M, Wühl E, Jourdan C (2005). Altered morphologic properties of large arteries in children with chronic renal failure and after renal transplantation. J Am Soc Nephrol.

[4] Shroff R, Weaver DJ Jr, Mitsnefes MM (2011). Cardiovascular complications in children with chronic kidney disease. Nat Rev Nephrol.

[5] Mitsnefes MM (2008). Cardiovascular complications of pediatric chronic kidney disease. *Pediatr Nephrol*.

[6] Kramer A, Boenink R, Stel VS (2021). The ERA-EDTA Registry Annual Report 2018: a summary. Clin Kidney J.

[7] Gerson AC, Wentz A, Abraham AG (2010). Health-related quality of life of children with mild to moderate chronic kidney disease. Pediatrics.

[8] Hamilton AJ, Caskey FJ, Casula A (2019). Psychosocial Health and Lifestyle Behaviors in Young Adults Receiving Renal Replacement Therapy Compared to the General Population: Findings From the SPEAK Study. Am J Kidney Dis.

[9] Kerr M, Bray B, Medcalf J (2012). Estimating the financial cost of chronic kidney disease to the NHS in England. Nephrol Dial Transplant.

[10] NHS Blood and Transplant (2009). Cost-effectiveness of transplantation. https://nhsbtmediaservices.blob.core.windows.net/organ-donation-assets/pdfs/Organ_Donation_Registry_Fact_Sheet_7_21337.pdf.

[11] Chadban SJ, Ahn C, Axelrod DA (2020). KDIGO Clinical Practice Guideline on the Evaluation and Management of Candidates for Kidney Transplantation. Transplantation.

[12] Van Stralen KJ, Harambat J, Clayton P (2016). Demographics of CKD and ESRD in children. Pediatric Kidney Disease.

[13] Plumb L, Boother EJ, Caskey FJ (2020). The incidence of and risk factors for late presentation of childhood chronic kidney disease: A systematic review and meta-analysis. PLoS One.

[14] Pruthi R, Casula A, Inward C (2016). Early Requirement for RRT in Children at Presentation in the United Kingdom: Association with Transplantation and Survival. *CJASN*.

[15] Scott SE, Walter FM, Webster A (2013). The model of pathways to treatment: conceptualization and integration with existing theory. Br J Health Psychol.

[16] (2022). UK renal registry 25th annual report – data to 31/12/2021. https://ukkidney.org/sites/renal.org/files/publication/file-attachments/25th%20Annual%20Report%20Final%2030.6.23%20-%20Edited%202024.pdf.

[17] Clarke V, Braun V, Hayfield N (2015). Thematic analysis. Qualitative psychology: A practical guide to research methods.

[18] Strauss A, Corbin JM (1997). Grounded Theory in Practice.

[19] Tong A, Sainsbury P, Craig J (2007). Consolidated criteria for reporting qualitative research (COREQ): a 32-item checklist for interviews and focus groups. Int J Qual Health Care.

[20] Tjaden L, Tong A, Henning P (2012). Children’s experiences of dialysis: a systematic review of qualitative studies. Arch Dis Child.

[21] Tong A, Morton R, Howard K (2009). Adolescent experiences following organ transplantation: a systematic review of qualitative studies. J Pediatr.

[22] Tong A, Henning P, Wong G (2013). Experiences and perspectives of adolescents and young adults with advanced CKD. Am J Kidney Dis.

[23] Olausson B, Utbult Y, Hansson S (2006). Transplanted children’s experiences of daily living: children’s narratives about their lives following transplantation. Pediatr Transplant.

[24] Tong A, Morton R, Howard K (2011). “When I had my transplant, I became normal.” Adolescent perspectives on life after kidney transplantation. Pediatr Transplant.

[25] Simons LE, McCormick ML, Mee LL (2009). Parent and patient perspectives on barriers to medication adherence in adolescent transplant recipients. Pediatr Transplant.

[26] Plumb L, Sinha MD, Jones T (2025). Identifying children who develop severe chronic kidney disease using primary care records. PLoS One.

[27] Zola IK (1973). Pathways to the doctor—From person to patient. *Social Science & Medicine (1967)*.

[28] Andersen RS, Paarup B, Vedsted P (2010). ‘Containment’ as an analytical framework for understanding patient delay: A qualitative study of cancer patients’ symptom interpretation processes. *Social Science & Medicine*.

[29] Teasdale EJ, Leydon G, Fraser S (2017). Patients’ Experiences After CKD Diagnosis: A Meta-ethnographic Study and Systematic Review. Am J Kidney Dis.

[30] Yoos HL, Kitzman H, McMullen A (2005). The language of breathlessness: do families and health care providers speak the same language when describing asthma symptoms?. J Pediatr Health Care.

[31] Dixon-Woods M, Findlay M, Young B (2001). Parents’ accounts of obtaining a diagnosis of childhood cancer. The Lancet.

[32] Holm KE, Patterson JM, Gurney JG (2003). Parental involvement and family-centered care in the diagnostic and treatment phases of childhood cancer: results from a qualitative study. J Pediatr Oncol Nurs.

[33] Granier S, Owen P, Pill R (1998). Recognising meningococcal disease in primary care: qualitative study of how general practitioners process clinical and contextual information. BMJ.

[34] Shanmugavadivel D, Walker D, Liu J-F (2016). HeadSmart: are you brain tumour aware?. Paediatr Child Health (Oxford).

[35] Calderon-Margalit R, Golan E, Twig G (2018). History of Childhood Kidney Disease and Risk of Adult End-Stage Renal Disease. N Engl J Med.

[36] Greenberg JH, Coca S, Parikh CR (2014). Long-term risk of chronic kidney disease and mortality in children after acute kidney injury: a systematic review. BMC Nephrol.

[37] Sangla A, Kandasamy Y (2021). Effects of prematurity on long-term renal health: a systematic review. BMJ Open.

[38] (2021). Chronic Kidney Disease: Evidence Reviews for Children and Young People Who Should Be Tested for CKD (National Institute for Health and Care Excellence).

