# Supplementary material for: Examining the pathway to specialist care for children and young people with late presentation of chronic kidney disease in the UK: a qualitative study
Source: BMJ Open. 2025 Nov 9;15(11):e096266. doi: 10.1136/bmjopen-2024-096266 (PMC12598940; doi:10.1136/bmjopen-2024-096266)
Supplement: online supplemental file 1 [file bmjopen-15-11-s001.pdf]

UnPiCK study Phase One: Interview topic guide.

1. Confirm understanding of study, participation, recording and confidentiality.
2. Take informed consent.

Introduction

Thank you again for agreeing to take part in this study. I'd like to start by reminding you that everything you say is confidential to me and my research supervisors. Quotes from this interview, if used, will have all personal details removed before they are included in reports. This interview is to gain an insight into your views and experiences as a patient/parent/carer: there aren't right or wrong answers.

Demographic questions (social context):

To start, could I ask a few basic questions about you and your background? These questions are not intended to be judgemental, but will help me to understand how what we discuss fits into the context of your family, work and social life.

1. What is your age (carers only): 18-24 25-34 35-44 45-54 65-74 >75 years?
2. What is your ethnicity?
3. Are you employed? If yes, what is your occupation?
4. Who lives with you at home?
5. Do you have a family history of kidney problems?
6. Can you tell me what your (child's) kidney condition is? When was it diagnosed?
7. (Child only): Do you have any (other) medical conditions? What are they?

Pathway to diagnosis (based on Model of Pathways to Treatment)*

1. Opening statement: I'd like to talk about your experience of finding out you (or your child) had a kidney condition, right from the first time you became aware that something wasn't quite right. From the beginning, can you tell me in your own words, your story of what has happened?
2. [If symptoms reported] Can you describe these symptoms a little more? *[Prompt: location, length of time, severity, ever had this before?]*
3. Who noticed the symptom(s) first? *[Prompt: them/child/parent/friend/teacher/other.*
4. When you first noticed the symptom(s) what did you think was the cause? How did it make you feel? Did you continue to feel like this? *[Prompt: if any change in mind-set, why? When?]* [Parent- Did the problem affect your child in any way? If yes, how?]
5. Did you discuss your/your child's symptoms with anyone? *[Prompt: Who were they? What did they say? Did you tell more than one person, if so what did they say? Did the encourage/discourage medical review?]*
6. Did you look anywhere else for information? *[Prompt: internet, books, radio, tv]*
7. Was there anything else happening that made you concerned there might be a problem?
8. When did you first see a doctor about the symptom(s)? *[Prompt: or another medical professional]*
9. What made you decide to see someone about the symptom(s) *[Prompt: trigger]*

10. After you had decided to see a doctor, was there anything that stopped you from going? *[Prompt: Was there any delay for any reason?]*
11. Were you seen by a doctor as soon as you would have liked? *[Access issues]*
12. When you went to see the doctor, did you have an idea about what might be wrong? *[Prompt: Do you know what made you think this?]*
13. Tell me about the appointment with the doctor *[Prompt: did it go well/badly and if so why? Did they perform any tests: urine/BP/bloods/weight? What was their diagnosis/advice? Did they suggest a follow-up review?]*
14. What happened after the visit to your doctor? *[Prompt: watch and wait, reassurance, referral to hospital?]*
15. How did you feel? *[Prompt: what made you feel that way?]* [Parent- how did your child react?]
16. When did you see a doctor next and why? **[Repeat questions 15-17 for repeated visits]**

Kidney diagnosis

1. At what point did someone mention that there might be a problem with the kidneys? *[Prompt: Where was this? When? Who was it?]*
2. What were your initial thoughts? *[Prompt: Surprise/upset/grief/anger?]*
3. How did they explain this to you? *[Prompt: Did you get the impression this was a big/long-term problem or something temporary? What did they say that made you think this?]*
4. What happened after this?
5. Tell me about the first time you saw a kidney doctor/specialist *[Prompt: Where was it? When? Who? What did they say? What happened?]*
6. How did that make you feel? [Parent- how did your child react?
7. Can you tell me about the first time you found out you (or your child) would need to start dialysis? *[Prompt: What had happened before this point? Who told you? Where? When?]*
8. How did that make you feel? [Parent- how did your child react?]

Impact of late presentation

1. What are your thoughts/feelings on the whole experience **now**? Of finding out about the kidney condition? [Parent- how does your child feel?]
2. How has life changed for you since the diagnosis? *[Prompt: school, work, how has your daily routine changed, has it affected family members? Has it changed anything for the better?]*
3. In your opinion, do you feel there was anything that prolonged you finding out your child had a kidney condition? *[Prompt: Difficulty recognising symptoms, issues with getting an appointment, stressors from work/family, medical staff attitudes/knowledge]*
4. Have you any thoughts about why you (or your child) developed a kidney problem?
5. Did you know anyone who had kidney problems before you (or your child) was diagnosed? *[Prompt: Who? What is the kidney problem? What was your understanding of that problem?]*
6. What would you have expected the signs/symptoms of a kidney problem to be?
7. Is there anything you now know that would have been helpful to know before you (or your child) was diagnosed? *[Prompts: symptoms, blood test]*

Conclusion

- We’ve been through a lot today, but is there anything else you would like to discuss?
- Are there any questions you would like to ask me?

Thanks, confirmation of confidentiality.

* Reference: Scott S, Walter F, Webster A, Sutton S, Emery J. The model of pathways to treatment: conceptualization and integration with existing theory. British journal of health psychology. 2013; 18(1):45-65.
