# Supplementary material for: Examining the pathway to specialist care for children and young people with late presentation of chronic kidney disease in the UK: a qualitative study
Source: BMJ Open. 2025 Nov 9;15(11):e096266. doi: 10.1136/bmjopen-2024-096266 (PMC12598940; doi:10.1136/bmjopen-2024-096266)
Supplement: Supplementary file 2 [file bmjopen-2024-096266.reviewer_comments.pdf]

PEER REVIEW HISTORY

BMJ Open publishes all reviews undertaken for accepted manuscripts. Reviewers are asked to complete a checklist review form and are provided with free text boxes to elaborate on their assessment. These free text comments are reproduced below.

ARTICLE DETAILS

Title (Provisional)

Examining the pathway to specialist care for children and young people with late presentation of chronic kidney disease in the UK: a qualitative study

Authors

Plumb, Lucy; Sinha, Manish; Ridd, Matthew J; Caskey, Fergus; Ben-Shlomo, Yoav; Owen-Smith, Amanda

---

VERSION 1 - REVIEW

Reviewer **1**

Name **Trachtman, Howard**

Affiliation **Division of Nephrology, Department of Pediatrics, University of Michigan, Ann Arbor, Michigan, USA**

Date **10-Mar-2025**

COI **None**

This is a very interesting paper and unfortunately I suspect it addressesa problem that is more common than nephrologists acknowledge. The approach seems appropriate and the conclusions are very modest and justified. The following are some suggestions that might improve the manuscript

1. CKD is a rare condition in pediatrics and it would be worth addressing the impact of that fact alone on the patient/family path to seeking attention and establishing a diagnosis

2. Earlier stage CKD can be quite silent. But CKD stage 4 when patients need RRT within 90 days like in this study is usually symptomatic. It would be helpful to give more detail in the symptom or the cluster of symptom that triggered recognition of the kidney disease

3. More data on the children including height, weight, and medications would be of interest

4. It would be of interest to provide more detail on the parents/care givers and their life situation to better understand how they pursued their child's diagnosis.

5. It seems as if one patient had HUS. This an abrupt condition that lends itself to a rapid diagnosis. Were there other cases of abrupt onset of kidney disease? If so, it would be

important to distinguish them from conditions like CAKUT that might follow a more indolent course with a risk of delayed diagnosis.

6. It would be helpful to provide some discrete recommendations based on the findings that could be tested in other populations at risk of CKD

**Reviewer**	**2**
**Name**	**Evers, Katrina**
**Affiliation**	**University Children's Hospital Basel, Pediatric Nephrology**
**Date**	**11-Aug-2025**
**COI**	**None**

The authors have focused on a very common and frequently neglected topic with children and adolescents presenting late with chronic kidney disease. The study is solid and it is represented here in a clear and structured way. It is very important to raise awareness for chronic kidney disease, because not only lay people cannot read the symptoms but very often also colleagues in primary care do not see an association with certain symptoms and lab findings. It is furthermore very interesting to get insight into memories and thoughts of affected families. The authors have done a very good job, spinning the red thread and also keeping the reader‘s attention.

VERSION 1 - AUTHOR RESPONSE

Reviewer #1		
	This is a very interesting paper and unfortunately, I suspect it addresses a problem that is more common than nephrologists acknowledge. The approach seems appropriate and the conclusions are very modest and justified.	Thank you for your kind comments and for reviewing our paper. The manuscript now stands at 3175 words length.
1	CKD is a rare condition in paediatrics and it would be worth addressing the impact of that fact alone on the patient/family path to seeking attention and establishing a diagnosis	Thank you. We already refer to the rarity of childhood CKD and as such the challenge in raising awareness among families with the following discussion text:<br><br>*"For patients who experienced a long appraisal period, interventions which improve lay knowledge of kidney disease and associated symptomatology may help reduce this interval. Awareness of kidney disease and function among the general population is recognised to be low[33] and was cited as a barrier to diagnosis by participants*

		in this study. Raising awareness of symptoms associated with kidney function decline could arm families with the requisite knowledge to consider timelier medical review, particularly in light of a child's age and other risk factors. Given the rarity of childhood CKD, it is unclear whether such campaigns would prove successful in improving timely detection; however a similar campaign for childhood brain tumours was successful in reducing the total diagnostic interval[34], suggesting this may be feasible."
2	Earlier stage CKD can be quite silent. But CKD stage 4 when patients need RRT within 90 days like in this study is usually symptomatic. It would be helpful to give more detail in the symptom or the cluster of symptom that triggered recognition of the kidney disease	Thank you for this comment. Symptoms that were spontaneously reported by parent and CYP participants and their frequency are now highlighted in table 3 with illustrative quotes.
3	More data on the children including height, weight, and medications would be of interest	Unfortunately, we were asked to limit the amount of social and clinical data collected by the research ethics committee who approved our application; therefore height/weight and medication data are unavailable.
4	It would be of interest to provide more detail on the parents/care givers and their life situation to better understand how they pursued their child's diagnosis.	Thank you. We were able to capture information about the child's family and the parents employment as part of the qualitative interviews – these are now added to table 2 for the reader's information.
5	It seems as if one patient had HUS. This an abrupt condition that lends itself to a rapid diagnosis. Were there other cases of abrupt onset of kidney disease? If so, it would be important to distinguish them from conditions like CAKUT that might follow a more indolent course with a risk of delayed diagnosis.	Thank you for this comment. We have now added a table to enable comparison of each child's diagnosis with the pattern of presentation (table 5). We also expanded the discussion to refer to patterns of presentation in relation to kidney disease diagnosis, with the following text:<br><br>*" In the sample of families interviewed, acute presentation represented acute kidney injury which failed to recover, leading to dialysis dependence. Most children experiencing an acute or semi-acute presentation had underlying glomerular or unknown kidney disorders, which may represent rapidly progressive disease that was not amenable to intervention. However, we also noted that*

		children with these conditions also experienced other patterns of presentation also, emphasising the importance of symptom recognition and targeted kidney testing when these are present."
6	It would be helpful to provide some discrete recommendations based on the findings that could be tested in other populations at risk of CKD	Thank you. In the discussion, we already refer to consideration of targeted testing in light of underlying risk factors but have expanded on this to consider symptoms and age as factors in this process:<br><br>*"However, three children had potential risk factors for the development of CKD in the form of an underlying syndromal disorder, AKI and prematurity respectively, raising the question of whether targeted monitoring of those in known 'at risk' categories may have led to earlier diagnosis and intervention. Similarly, as the incidence of kidney failure (and thus late presentation) is highest in adolescence[14,36], consideration of targeted kidney testing in the context of a child's age when key symptoms are present may support earlier detection."*<br><br>Since submission of this paper, a quantitative study from our group looking at the presence and predictive value of symptoms presenting to primary care before a diagnosis of advanced kidney disease has been published and so we have referred to this, which supports the qualitative findings of this study (reference 26).
Reviewer #2		
	The authors have focused on a very common and frequently neglected topic with children and adolescents presenting late with chronic kidney disease. The study is solid and it is represented here in a clear and structured way. It is very important to raise awareness for chronic kidney disease, because not only lay people cannot read the symptoms but very often also colleagues in primary care do not see an association with certain symptoms and lab findings. It is furthermore very interesting to get insight into memories and thoughts of affected families. The authors have done a very good	Thank you for your considerate comments and for your review.

	job, spinning the red thread and also keeping the reader‘s attention.	
